# Measuring multisensory integration: from reaction times to spike counts

**DOI:** 10.1038/s41598-017-03219-5

**Published:** 2017-06-08

**Authors:** Hans Colonius, Adele Diederich

**Affiliations:** 1 Carl von Ossietzky Universität Oldenburg, Department of Psychology, Oldenburg, 26111 Germany; 2 Jacobs University Bremen, Life Sciences & Chemistry, Bremen, 28759 Germany

## Abstract

A neuron is categorized as “multisensory” if there is a statistically significant difference between the response evoked, e.g., by a crossmodal stimulus combination and that evoked by the most effective of its components separately. Being responsive to multiple sensory modalities does not guarantee that a neuron has actually engaged in integrating its multiple sensory inputs: it could simply respond to the stimulus component eliciting the strongest response in a given trial. Crossmodal enhancement is commonly expressed as a proportion of the strongest mean unisensory response. This traditional index does not take into account any statistical dependency between the sensory channels under crossmodal stimulation. We propose an alternative index measuring by how much the multisensory response surpasses the level obtainable by optimally combining the unisensory responses, with optimality defined as probability summation under maximal negative stochastic dependence. The new index is analogous to measuring crossmodal enhancement in reaction time studies by the strength of violation of the “race model inequality’, a numerical measure of multisensory integration. Since the new index tends to be smaller than the traditional one, neurons previously labeled as “multisensory’ may lose that property. The index is easy to compute and it is sensitive to variability in data.

## Introduction

Single neurons in the deep layers of the mammalian superior colliculus (SC) integrate afferent visual, auditory, and somatosensory cues and generate efferent motor commands to structures innervating the musculature of, e.g., the eyes and hands^1, 2^. In a recent, multi-authored paper^3^ *multisensory integration* has been defined operationally “… as the neural process by which unisensory signals are combined to produce a multisensory response that is significantly different from the responses evoked by the modality-specific component stimuli”^3^, p. 1719. A related paper^4^ illustrates the standard method for identifying and evaluating the computations underlying multisensory integration by the following example. At the level of a single SC neuron, response strength has traditionally been measured by the absolute number of impulses (spikes) registered within a fixed time interval after stimulus presentation (or, sometimes, by the firing rate within this interval). A neuron is categorized as “multisensory” if the average absolute number of spikes to a crossmodal stimulus combination in a given sample of recordings is statistically significantly higher compared to the higher average absolute number of spikes to a unisensory stimulus. Analogously, in case of inhibition, “multisensory” means that the average absolute number of spikes to a crossmodal stimulus combination is significantly lower compared to the lower average absolute number of spikes to a unisensory stimulus^2, 5^. Moreover, if a neuron responds, for example, to visual but not to auditory stimulation and if the response to a visual-auditory combination differs (in the above sense) from the response to the visual stimulus, it is also considered being “multisensory”.

Once multisensory enhancement or inhibition has been identified, the computational mode can be further subdivided into *superadditive*, *additive*, or *subadditive* enhancement by comparing the multisensory response to the predicted sum of the unisensory responses^4^. Specifically, according to the “inverse effectiveness rule” of multisensory integration^2^ the operating mode of a multisensory response is super-additive for weak intensity stimuli and transforms into an additive or even sub-additive combination for more intense stimuli^6^.

Up to date, the most widely used descriptive measure of the magnitude of multisensory integration is the *crossmodal enhancement index* (CRE), also termed *crossmodal interaction index*. It is defined as

1 $${\rm{CRE}}=\frac{{\rm{CM}}-{{\rm{SM}}}_{{\rm{\max }}}}{{{\rm{SM}}}_{{\rm{\max }}}}\times 100,$$

where, at the sample level, CM is the mean number of spikes in response to the crossmodal stimulus and SM_max_ is the mean number of spikes to the most effective modality-specific component stimulus^7^. Thus, CRE expresses crossmodal enhancement as a proportion of the strongest unisensory response.

Some modifications of CRE have been proposed as well^8^. Prominently, in the “additive model”, term SM_max_ in Equation (1) is replaced by the sum of the unisensory responses^9^. The additive version has raised some controversy because observing a multisensory response larger than the largest unisensory response but smaller than the sum might be misinterpreted as response inhibition^4^. In summary, the issue of exactly how to measure the strength of multisensory interactions has been under debate for some time^4, 8, 10^.

The starting point of a new measure developed here is the observation that being responsive to multiple sensory modalities does not guarantee that a neuron has actually engaged in integrating its multiple sensory inputs, rather than simply responding to the most effective stimulus in a given trial, i.e., to the stimulus eliciting the strongest response. As Stein and colleagues^4^ (ibid, p. 114) have put it, “*At the time of the early physiology studies in the 1980s, it was considered possible that these neurons only represented a common route by which independent inputs from a variety of senses could gain access to the same motor apparatus in generating behavior (e.g., possibly employing a “winner-take-all” algorithm*).”

In other words, it is possible that the response to a bimodal stimulus is simply determined by the larger of the responses to the modality-specific components in any given trial, e.g., by the the component that happens to elicit the higher absolute number of spikes in a given trial. Assuming random variation of the responses, such a mechanism is known as *probability summation*. It would not be considered “true” multisensory integration as it does not actually combine the activities elicited by the modality-specific stimulus components (“coactivation”). Let us assume, for a moment, that such a probability summation mechanism actually generates the multisensory responses. Intriguingly, it will be shown below that the expected value (in a sample: the average) of the random number of spikes elicited by a crossmodal stimulus combination will be maximal when probability summation operates under (maximal) negative statistical dependency, i.e., when large responses to one stimulus component tend to co-occur with small responses to the other component, and vice versa. The measure to be presented below takes this maximum as a benchmark.

Given that the actual computations performed by a multisensory neuron are still not fully understood^11^, developing a new measure should not depend on specific assumptions about the multisensory integration process. Note that it is not claimed here that the neuron actually operates under this negative dependence rule. As long as probability summation is considered a viable alternative to “true” multisensory integration, however, some specification of the stochastic relation between the unisensory responses has to be made. Assuming maximal negative dependency is simply the most conservative choice. Whenever there is empirical or theoretical evidence in favor of some other form of dependence, e.g. stochastic independence, this could be taken as benchmark as well. Because, in general, the new measure is more restrictive than the traditional CRE, many neurons previously categorized as “multisensory” risk losing that property. The purpose of the new measure corresponds to that of the traditional measure: given a fixed statistical criterion, one may categorize a single neuron as either being “multisensory” or not. It is of course possible that a neuron actually “truly” integrates the unimodal activations but still does not meet the criterion set by maximal negative probability summation. However, as long as one has no direct insight into the integration mechanism, an alternative interpretation in terms of probability summation simply cannot be ruled out. Moreover, when such a criterion is not met, the value of the new measure can also be taken as an indicator of the strength of multisensory integration occurring. It should be noted, however, that a more liberal definition of a multisensory neuron is possible, according to which “any neuron that responds to or is influenced by stimuli from two or more sensory modalities”^12^ would be considered “multisensory”.

In order to gain more insight into the new definition, we first consider an established measure of crossmodal enhancement in behavioral data, the race model inequality for reaction times. A numerical measure derived from that inequality turns out to be completely analogous to the measure proposed here for neural data. Then, after introducing the new index, its properties are illustrated on a sample of spike count data (Mark Wallace, personal communication, July 18, 2015) and compared to the traditional index. In addition, the special parametric case of Poisson-distributed spikes serves to demonstrate that, in contrast to the traditional index, the new one takes the variability of the data into account.

## Measuring crossmodal enhancement of reaction time

In the *redundant signals paradigm*, stimuli from two (or more) different modalities are presented simultaneously, and participants are instructed to respond to a stimulus of any modality, whichever is detected first. Besides comparing relative detection frequencies of unimodal vs. crossmodal stimuli, behavioral response strength is most often measured by reaction time (RT), that is, the time it takes a participant to respond (e.g., via button press) to a suddenly appearing stimulus, often visual or acoustic. Typically, time to respond in the crossmodal condition is shorter than that in either of the unimodal conditions. In analogy to CRE at the neural level, the index of *crossmodal response enhancement for reaction time* (CRE_RT_) is traditionally defined as^13–16^

2 $${{\rm{CRE}}}_{{\rm{RT}}}=\frac{R{T}_{\min }-R{T}_{CM}}{R{T}_{\min }}\times \mathrm{100,}$$

where *RT*_*CM*_ is the mean RT to the crossmodal stimulus and *RT*_min_ is the faster of the mean RTs to the modality-specific stimuli. Thus, CRE_RT_ expresses multisensory enhancement as a proportional reduction of the faster unisensory response by the crossmodal response. For concreteness, we rewrite CRE_RT_ at the population level, for the case of visual-auditory stimulation, with E*RT*_*V*_, E*RT*_*A*_, and E*RT*_*VA*_ denoting expected reaction time to the visual stimulus, the auditory stimulus, or the visual-auditory stimulus combination, respectively. CRE_RT_ then becomes

3 $${{\rm{CRE}}}_{{\rm{RT}}}=\frac{\min \,\{{\rm{E}}R{T}_{V},{\rm{E}}R{T}_{A}\}-{\rm{E}}R{T}_{VA}}{\min \,\{{\rm{E}}R{T}_{V},{\rm{E}}R{T}_{A}\}}\times 100,$$

Just as neural measure CRE of Equation (1), index CRE_RT_ has descriptive value. For example, CRE_RT_ = 10 means that response to the visual-auditory stimulus is 10% faster than the faster of the expected response times to unimodal visual and auditory stimuli.

However, it has been recognized early on ref. 17 that simply comparing mean RTs to crossmodal and unimodal stimuli is not diagnostic with respect to a presumed underlying multisensory integration process, for the following reason. Let us assume that in the crossmodal condition, (i) each individual stimulus elicits a process performed in parallel to the others and, (ii), the finishing time of the faster process determines the observed RT. This is known as the “race model” for RTs. Assuming random variability of the finishing times, the mean RT in the crossmodal condition is predicted to be shorter than the faster of the unimodal mean RTs. This is an effect of probability summation and no “true” multisensory integration of the unisensory processes takes place. It has also been called “statistical facilitation” in this context.

In order to gauge whether observed crossmodal RTs are faster than predicted by statistical facilitation, Jeff Miller^18, 19^ proposed an even stronger test, the race model inequality (RMI) test,

$$P(\min \,\{V,A\}\le t)\le P(V\le t)+P(A\le t)$$

or

4 $${F}_{VA}(t)\le {F}_{V}(t)+{F}_{A}(t)\,{\rm{for}}\,{\rm{all}}\,\,t,\,t\ge 0.$$

Here *V* and *A* denote visual and auditory processing times, respectively, with *F*_*V*_, *F*_*A*_ the corresponding unimodal RT distributions, and *F*_*VA*_ the distribution of the RTs in the crossmodal (visual-auditory) condition. Violation of Equation (4) at any time point *t* is evidence in favor of some form of multisensory integration taking place above statistical facilitation, often termed “coactivation”. Note that stochastic independence between the processing times *V* and *A* is not required, but the test is valid only if an assumption of “context independence” holds: the distributions of *V* and *A* in the unimodal conditions must equal their corresponding marginal distributions in the crossmodal condition^20, 21^ (see next subsection).

The race model inequality has become the standard tool for testing whether observed reaction times to crossmodal stimuli are faster than predicted by a simple statistical facilitation mechanism. Gondan and Minakata^22^ report 83 studies from 2011 to 2014 performing the inequality test using a variety of statistical methods. Because, unlike CRE, Inequality (4) does not represent a single numerical measure of the amount of crossmodal enhancement, it has become practice to compute the following geometric measure: the area *S* between *F*_*VA*_ and *F*_*V*_ + *F*_*A*_ defined by all *t* values where the race model inequality is violated:

5 $$S={\int }_{0}^{\infty }{{\bf{1}}}_{C}(t)\,dt$$

with

$$C=\{t\,:\,{F}_{VA}(t) > {F}_{V}(t)+{F}_{A}(t)\},$$

with indicator function **1**_*C*_(*t*) taking the value of 1 if *t* ∈ *C* and zero otherwise. The sample estimate of area *S* is then taken as index of the strength of violation of the inequality. Notably, a brief discussion of the race model inequality in the next section reveals that area *S* can be interpreted as the expected value of random variable min{*V*, *A*} (under maximal negative dependence) and estimating *S* is rather straightforward not requiring any geometric argument (for details, see also ref. 23).

### Context independence and coupling of random variables

Sometimes, instead of Equation (4), a more restrictive inequality is tested,

6 $${F}_{VA}(t)\le {F}_{V}(t)+{F}_{A}(t)-{F}_{V}(t)\times {F}_{A}(t),$$

Assuming *stochastic independence* between *V* and *A*. This raises the general question of how the random variables in the unimodal conditions, *V* and *A*, related. Actually, as already observed by R.D. Luce^20^, p. 130, there exists –a-priori– *no stochastic relation* between them: the probability measures for *V* and *A*, *P*_*V*_ and *P*_*A*_, are defined on different probability spaces, thus *V* and *A* are stochastically unrelated: there is no empirical context (e.g., trial number) in which a unimodal event {*V* ≤ *s*} co-occurs with a unimodal event {*A* ≤ *t*} to define a joint distribution for (*V*, *A*). Nevertheless, such a joint distribution can always be constructed by the stochastic concept of *coupling*. A *coupling* of random variables *V* and *A* is a pair of random variables $(\hat{V},\hat{A})$ with a bivariate distribution function *H*_*VA*_(*s*, *t*) such that its marginal distributions are identical to *F*_*V*_ and *F*_*A*_ respectively, i.e.,

$$V\mathop{=}\limits^{d}\hat{V}\,\,{\rm{and}}\,A\mathop{=}\limits^{d}\hat{A},$$

where $\mathop{=}\limits^{d}$ means “equality-in-distribution”. Thus, existence of a coupling is equivalent to the assumption of “context independence” mentioned above. Inequality (6) corresponds to an *independent coupling* of *V* and *A* with

$${H}_{VA}(s,t)={F}_{V}(s)\times {F}_{A}(t),$$

But there exists an infinite number of possible couplings (for a comprehensive treatment of the theory of coupling, see ref. 24).

For the race model Inequality (4), which can be written equivalently as

$${F}_{VA}(t)\le \,\min \,\{{F}_{V}(t)+{F}_{A}(t),\,\mathrm{1\}},t\ge 0,$$

It turns out that the right-hand side corresponds to the coupling of *V* and *A* generating maximal negative stochastic dependence between the two random variables. Moreover, the area *S* between the distribution functions *F*_*VA*_ and min{*F*_*V*_(*t*) + *F*_*A*_(*t*), 1} equals the expected value of random variable min{*V*, *A*}, i.e.,

$$S={{\rm{E}}}^{-}\,\min \,\{V,A\},$$

Under maximal negative dependence between *V* and *A*, with superscript “—” indicating maximal negative dependence.

### CRE of RT under maximal negative dependence

A standard scenario for negative dependence of the processing times is “limited capacity”: in any given trial, participant may focus attention on one sensory modality and, because of limited attentional capacity, processing of the other modality may become slower. A measure of crossmodal response enhancement for reaction times, based on maximal negative dependence, can then be defined by replacing min{E*RT*_*V*_, E*RT*_*A*_} in Equation (3) by area *S*, yielding:

7 $${{\rm{CRE}}}_{{\rm{RT}}}^{-}=\frac{{{\rm{E}}}^{-}\,\min \,\{V,A\}-{\rm{E}}R{T}_{VA}}{{{\rm{E}}}^{-}\,\min \,\{V,A\}}\times 100.$$

Because ${{\rm{E}}}^{-}\,\min \,\{V,A\}\le \,\min \,\{{\rm{E}}R{T}_{V},{\rm{E}}R{T}_{A}\}$, a direct consequence of applying *Jensen’s inequality* (see, e.g., ref. 25 p. 51), it follows from comparing Equations (3) and (7) that always

$${{\rm{CRE}}}_{{\rm{RT}}}^{-}\le {{\rm{CRE}}}_{{\rm{RT}}}.$$

In other words, the new index of crossmodal response enhancement for RT is more conservative than the traditional one. Proof of all of the above statements, being analogous to the one given for spike counts in the next section, is omitted here, but see refs 21, 26 and 27.

## Measuring crossmodal enhancement in single neurons

Going over from reaction times to spike counts in single neurons involves two major changes. First, instead of measuring a continuous random variable (RT), the discrete number of spikes emitted in a given time interval by a neuron is the random variable of interest. Second, the minimum reaction time as unimodal reference point is replaced by the maximum spike count of the unisensory responses (within a given time interval) of the neuron.

To fix ideas, let *N*_*V*_, *N*_*A*_, and *N*_*VA*_ denote the random number of impulses (spikes), following unisensory (visual, auditory) and crossmodal (visual-auditory) stimulation, respectively, without assuming any specific parametric distribution for these random variables. Inserting their expected values into the traditional CRE of Equation (1) yields

8 $${{\rm{CRE}}}_{{\rm{SP}}}=\frac{{\rm{E}}{N}_{VA}-\,\max \,\{{\rm{E}}{N}_{V},{\rm{E}}{N}_{A}\}}{\max \,\{{\rm{E}}{N}_{V},{\rm{E}}{N}_{A}\}}\times 100,$$

where subscript SP indicates measurement of spikes. At the level of samples, the expected values are replaced by arithmetic averages.

Realizations of random variables *N*_*V*_ and *N*_*A*_, with distribution functions *G*_*V*_ and *G*_*A*_, respectively, are collected across experimental trials under different stimulus conditions (modality-specific and crossmodal). Thus, as observed above for reaction times, they refer to distinct probability spaces and there is –a-priori– no natural way to combine the results from modality-specific visual and auditory trials. In particular, any assumption about stochastic (in-)dependence between *N*_*V*_ and *N*_*A*_ is void. Nevertheless, one can define a stochastic *coupling* of the two random variables. Coupling of *N*_*V*_ and *N*_*A*_ here amounts to defining a distribution *H*_*VA*_ for a bivariate random vector $({\tilde{N}}_{V},{\tilde{N}}_{A})$ in such a way that its marginal distributions are identical to *G*_*V*_ and *G*_*A*_.

Let ${H}_{VA}(m,n)=P({\tilde{N}}_{V}\le m,{\tilde{N}}_{A}\le n)$, $m,n=0,\mathrm{1,}\ldots$, be the distribution for some coupling of *N*_*V*_ and *N*_*A*_. As a bivariate (discrete) distribution, it obeys the Fréchet inequalities valid for any distribution^28^:

9 $$\max \,\mathrm{\{0},{G}_{V}(m)+{G}_{A}(n)-\mathrm{1\}}\le {H}_{VA}(m,n)\le \,\min \,\{{G}_{V}(m),{G}_{A}(n)\},$$

For all *m*, *n* = 0, 1, …. Setting *m* = *n*, we get

$${H}_{VA}(m,m)=P(\max \{{\tilde{N}}_{V},{\tilde{N}}_{A}\}\le m),$$

and from (9),

10 $$\begin{array}{rcl}{H}^{-}(m) & \equiv  & \max \,\mathrm{\{0},{G}_{V}(m)+{G}_{A}(m)-\mathrm{1\}}\\  & \le  & {H}_{VA}(m,m)\\  & \le  & \min \{{G}_{V}(m),{G}_{A}(m)\}\equiv {H}^{+}(m),\end{array}$$

For *m* = 0, 1, …. In (10) both upper bound *H* ^+^(*m*) and lower bound *H* ^−^(*m*) are univariate distribution functions of random variable $\max \,\{{\tilde{N}}_{V},{\tilde{N}}_{A}\}$. Moreover, it is well known^29^ that *H* ^+^ and *H* ^−^ represent distributions with maximal positive, respectively negative, dependence between ${\tilde{N}}_{V}$ and ${\tilde{N}}_{A}$, assuming non-degenerate marginal distributions *G*_*V*_ and *G*_*A*_. Going over to the means we obtain the following

**Proposition:** *Under any coupling of the univariate response random variables N*_*V*_ *and N*_*A*_, *the following bounds hold for expected value* Emax{*N*_*V*_, *N*_*A*_},

11 $$\max \,\{{\rm{E}}{N}_{V},{\rm{E}}{N}_{A}\}\le {\rm{Emax}}\{{N}_{V},{N}_{A}\}\le {{\rm{E}}}^{-}\,\max \,\{{N}_{V},{N}_{A}\},$$

*where* ${{\rm{E}}}^{-}\,\max \,\{{N}_{V},{N}_{A}\}$ *is the expected value under maximal negative dependence between the univariate response random variables*.

To prove the right-hand bound of the proposition, rewrite Equation (10) as

$$\begin{array}{c}1-{H}^{+}(m)\le 1-{H}_{VA}(m,m)=P(\max \,\{{N}_{V},{N}_{A}\} > m)\\ \quad \quad \quad \quad \quad \quad \quad \quad \quad \quad \quad \quad \le 1-{H}^{-}(m),\end{array}$$

For *m* = 0, 1, …. Summing over all *m* yields the result

$$\sum _{m=0}^{\infty }[1-{H}_{VA}(m,m)]={\rm{Emax}}\{{N}_{V},{N}_{A}\}\le {{\rm{E}}}^{-}\,\max \,\{{N}_{V},{N}_{A}\}.$$

The left-hand bound, $\max \,\{{\rm{E}}{N}_{V},{\rm{E}}{N}_{A}\}\le {\rm{Emax}}\{{N}_{V},{N}_{A}\}$ follows again from *Jensen’s inequality*.

### CRE in single neurons under maximal negative dependence

From Proposition 1 it is clear that the sample value of E^−^ max{*N*_*V*_, *N*_*A*_} is the largest mean obtainable from combining the unisensory responses via probability summation. Replacing max{*EN*_*V*_, *EN*_*A*_} by E^−^ max{*N*_*V*_, *N*_*A*_} in the traditional CRE_SP_ index of equation (8) results in the new index

12 $${{\rm{CRE}}}_{{\rm{SP}}}^{-}=\frac{{\rm{E}}{N}_{VA}-{{\rm{E}}}^{-}\,\max \,\{{N}_{V},{N}_{A}\}}{{{\rm{E}}}^{-}\,\max \,\{{N}_{V},{N}_{A}\}}\times 100.$$

This new index measures the degree by which a neuron’s observed multisensory response surpasses the level obtainable by optimally combining the unisensory responses (assuming that the neuron simply reacts to the more salient modality in any given crossmodal trial). The empirical test for multisensory enhancement then amounts to comparing the observed mean number of impulses to crossmodal stimulation with the estimate for E^−^max{*N*_*V*_, *N*_*A*_}. For empirical data, the expected value E*N*_*VA*_ is replaced by the sample mean of multisensory responses and E^−^ max{*N*_*V*_, *N*_*A*_} is estimated using the *method of antithetic variates* as demonstrated below (see also ref. 25).

#### Two important consequences

Applying the new index has two important consequences. First, given that the values of the new index are obviously always smaller or equal to the traditional index,

$${{\rm{CRE}}}_{{\rm{SP}}}^{-}\le {{\rm{CRE}}}_{{\rm{SP}}},$$

Some neurons previously labeled “multisensory” may lose that property under the new index. This is illustrated with an empirical data set following the next section.

Second, from the definition of CRE_SP_ it follows that changing the variability of the unisensory responses while leaving max{*N*_*V*_, *N*_*A*_} invariant, will not affect the value of the traditional crossmodal index. In contrast, the new index, being based on E^−^ max{*N*_*V*_, *N*_*A*_}, can be sensitive to such changes. This is illustrated here for the case of Poisson-distributed spikes.

#### Example: Poisson-distributed spikes

Let the spike counts *N*_*V*_ and *N*_*A*_ follow a Poisson distribution, i.e.,

13 $$P({N}_{i}=m)=\exp [-{\lambda }_{i}]\frac{{\lambda }_{i}^{m}}{m!}\,{\rm{for}}\,m=0,1,2\ldots .$$

with *i* = *V* or *i* = *A*. For this distribution, *EN*_*i*_ = *λ*_*i*_ and, for the variance, Var*N*_*i*_ = *λ*_*i*_ as well. Using the equality of E and Var, the traditional index can thus be rewritten as

14 $${{\rm{CRE}}}_{{\rm{SP}}}=\frac{{\rm{E}}{N}_{VA}-\,\max \,\{{\rm{Var}}{N}_{V},{\rm{Var}}{N}_{A}\}}{\max \,\{{\rm{Var}}{N}_{A},{\rm{Var}}{N}_{A}\}}\times 100,$$

We assume, without loss of generality, that Var*N*_*A*_ < Var*N*_*V*_. Obviously, increasing Var*N*_*A*_ will not change the value of CRE_SP_ as long as Var*N*_*A*_ is not strictly larger than Var*N*_*V*_. In contrast, as will now be shown, E^−^ max{*N*_*V*_, *N*_*A*_}, and therefore ${{\rm{CRE}}}_{{\rm{SP}}}^{-}$ as well, will not remain invariant with Var*N*_*A*_ increasing.

Inserting into the expected value yields

$$\begin{array}{c}{{\rm{E}}}^{-}\,\max \{{N}_{V},{N}_{A}\}=\sum _{m\mathrm{=0}}^{\infty }\mathrm{[1}-{H}^{-}(m)]\\ =\sum _{m=0}^{\infty }[1-\,\max \{0,\sum _{k=0}^{m}P({N}_{V}=k)+\sum _{k=0}^{m}P({N}_{A}=k)-1\}]\mathrm{.}\end{array}$$

For given values of parameters *λ*_*V*_ and *λ*_*A*_, approximate computation of this expected value is simplified by using the fact^30^ that the (cumulative) distribution for the Poisson is expressed in terms of the *incomplete gamma function*. Specifically, for i = *V*, *A*:

$$\sum _{k=0}^{m}P({N}_{i}=k)={\rm{\Gamma }}(m+\mathrm{1,}\,{\lambda }_{i})/{\rm{\Gamma }}(m\mathrm{).}$$

Here, the ratio ${\rm{\Gamma }}(m+1,{\lambda }_{i})/{\rm{\Gamma }}(m)$ is the *regularized incomplete gamma function* with ${\rm{\Gamma }}(m)=(m-\mathrm{1)!}$ and ${\rm{\Gamma }}(m,{\lambda }_{i})$ the *incomplete gamma function*

15 $$\Gamma (m,{\lambda }_{i})={\int }_{{\lambda }_{i}}^{\infty }{e}^{-t}{t}^{m-1}\,dt.$$

For illustration of the effect, we choose specific, but otherwise arbitrary, parameter values: E*N*_*VA*_ = 30 and, for Var*N*_*V*_ = *λ*_*V*_ = 22 and Var*N*_*V*_ = *λ*_*V*_ = 26, we varied Var*N*_*A*_ = *λ*_*A*_ between 5 and 22 and 26, respectively. Table 1 lists the corresponding values of CRE^−^_SP_ as a function of Var*N*_*V*_ and Var*N*_*A*_ as well as the CRE_SP_ for the two different values of Var*N*_*V*_. Notably, increasing Var*N*_*A*_ = *λ*_*A*_ corresponds to a strong decrease in CRE^−^_SP_, whereas CRE_SP_ remains invariant against such increase in variability of *N*_*A*_.

**Table 1:** Poisson-distributed spike counts: Values of CRE^−^_SP_ are shown as a function of *λ*_*A*_ = Var*N*_*A*_ and two fixed values of *λ*_*V*_ = Var*N*_*V*_. CRE^−^_SP_ decreases with increasing variability of *N*_*A*_, whereas CRE_SP_ remains constant.

λ_V_	λ_A_	CRE^−^_SP_	CRE_SP_
22	5	36.3	36.4
22	10	35.1	
22	16	29.0	
22	22	16.6	
26	5	15.4	15.4
26	10	15.0	
26	16	12.7	
26	22	6.3	
26	26	−0.2	

Note that Table 1 features the “inverse effectiveness” rule mentioned earlier. Increasing the *λ*_*V*_ intensity parameter of the Poisson distribution clearly results in decreasing both indexes, CRE^−^_SP_ and CRE_SP_. Moreover, CRE^−^_SP_ also decreases when the second parameter, *λ*_*A*_ increases toward the value of *λ*_*V*_. This prediction of the Poisson model is obviously empirically testable. This theoretical result is consistent with earlier results of R.C. Griffiths *et al*.^31^ about the correlation of Poisson random variables under maximal negative dependence. They found that, for a fixed value of *λ*_*V*_, say, maximal negative correlation increases (in absolute value) with *λ*_*A*_ getting closer to the value of *λ*_*V*_ (except for small non-mononicities due to the discreteness of the variables). This translates into E^−^ max{*N*_*V*_, *N*_*A*_} increasing as well, resulting in turn in smaller values of CRE^−^_SP_.

## Empirical data: absolute number of spikes

First, we demonstrate the computation of CRE^−^_SP_ and CRE_SP_ for a single-neuron data set, recordings from a cat *superior colliculus* (SC) neuron, followed by a comparison of both indexes on a larger number of such neurons. All data in this section has been obtained from the lab of Mark T. Wallace^12^ and represent a small sample of recordings published previously^32^. Since the data only serve for illustrating the approach, we limit methodological details to those necessary for understanding.

### Computing CRE^−^_SP_ and CRE_SP_ for data from a single neuron

The data set consists of the total number of spikes, recorded within a response window, that occurred from visual, auditory, and visual-auditory stimulation of one and the same neuron in *N* = 20 trials, respectively (details in Table 2). Neurons differ with respect to their spontaneous firing rate, i.e., spikes emitted that are not related to the stimulus presented (baseline firing). For a valid comparison of different multisensory neurons, spontaneous activity is usually removed. Spike numbers in the left-hand columns of Table 2 include spontaneous activity (S.A.), whereas the right-hand columns show the same recordings after S.A. was removed.

**Table 2:** Sample of recordings from a single cat SC: Columns 2 and 6 (**V**) are arranged by increasing order, 3 and 7 (**A**) by decreasing order.

trial	Spike numbers				Spike numbers w/o S.A.			
	V	A	max(V, A)	VA	V	A	max(V, A)	VA
1	3	8	8	11	1.1	7.5	7.5	18.9
2	4	8	8	22	2.1	7.5	7.5	13.3
3	5	7	7	17	3.1	6.5	6.5	15.9
4	5	7	7	19	3.1	6.5	6.5	14.9
5	5	7	7	18	3.1	6.5	6.5	9.9
6	6	7	7	13	4.1	6.5	6.5	14.9
7	6	6	6	18	4.1	5.5	5.5	7.9
8	7	6	7	11	5.1	5.5	5.5	22.9
9	7	6	7	26	5.1	5.5	5.5	16.9
10	8	6	8	20	6.1	5.5	6.1	24.9
11	8	6	8	28	6.1	5.5	6.1	15.9
12	9	6	9	19	7.1	5.5	7.1	21.9
13	9	5	9	25	7.1	4.5	7.1	11.9
14	10	5	10	15	8.1	4.5	8.1	13.9
15	10	5	10	17	8.1	4.5	8.1	15.9
16	10	4	10	19	8.1	3.5	8.1	15.9
17	11	4	11	19	9.1	3.5	9.1	14.9
18	11	4	11	18	9.1	3.5	9.1	27.9
19	13	4	13	31	11.1	3.5	11.1	13.9
20	14	4	14	17	12.1	3.5	12.1	7.9
mean	8.05	5.75	8.85	19.15	6.16	5.2	7.5	16.1
standard dev.	3.0	1.3	2.2	5.2	3.0	1.3	1.8	5.2

S.A. stands for “spontaneous activity” (4.26 spikes/s in this sample). Standard PSTHs (peristimulus time histograms) were computed. Spontaneous activity was computed from the 500 ms preceding each stimulus onset (allowing at least 1500 ms between each trial). A threshold of mean S.A. rate per 10 ms bin plus 2 standard deviations was computed, only used to determine onset and offset. Response onset was defined when the first spike occurred within the bin that rises above this threshold and remained above for at least 3 bins. Offset was counted as the last spike in the bin just before the response fell back below this threshold and remained below for 3 bins. The response window (duration) is the time between onset and offset. Total number of spikes (left columns in the table) include all spikes within the response window, which will inevitably include some S.A. The right columns include responses with S.A. removed. The expected number of S.A. spikes within the given window (i.e., S.A. times window size in seconds) was removed. This is never an integer and can sometimes cause negative values on some trials. This number represents “change from baseline firing” (information obtained from M. T. Wallace, personal communication, July 18, 2015).

Note that a-priori there is no fixed correspondence between trial number and the individual values of **V** and **A**. The antithetic variates method involves pairing the unisensory responses, sorted by increasing order (**V**) and by decreasing order (**A**), and computing **max**(**V**, **A**) for each pair. Their mean value represents an estimate of E^−^ max{*N*_*V*_, *N*_*A*_}, that is, of the maximum expected value from combining the unisensory responses achievable via negatively dependent probability summation. Note that the trial numbering of the **VA** values remains arbitrary, only the mean will be used in the computation.

Computing the traditional CRE_SP_ value by inserting the estimates from Table 2 in Equation (8), i.e., replacing the expected values by the means, yields

$$\begin{array}{c}{{\rm{CRE}}}_{{\rm{SP}}}=\frac{{\rm{E}}{N}_{VA}-\,\max \,\{{\rm{E}}{N}_{V},{\rm{E}}{N}_{A}\}}{\max \,\{{\rm{E}}{N}_{V},{\rm{E}}{N}_{A}\}}\times 100\\ \quad \quad \,\,\,\,\,\approx \,\frac{19.15-\,\max \,\mathrm{\{8.05},\mathrm{5.75\}}}{\max \,\mathrm{\{8.05},\mathrm{5.75\}}}\times 100=\mathrm{137.89[ \% ].}\end{array}$$

For spike numbers containing S.A. (left-hand columns). The corresponding value for the new index is estimated by inserting the estimates from Table 2 in Equation (12),

$$\begin{array}{c}{{\rm{CRE}}}_{{\rm{SP}}}^{-}=\frac{{\rm{E}}{N}_{VA}-{{\rm{E}}}^{-}\,\max \,\{{N}_{V},{N}_{A}\}}{{{\rm{E}}}^{-}\,\max \,\{{N}_{V},{N}_{A}\}}\times 100\\ \quad \quad \quad \approx \,\frac{19.15-8.85}{8.85}\times \mathrm{100=116.64[ \% ].}\end{array}$$

The corresponding values for responses with S.A. removed (right-hand columns) amount to

$${{\rm{CRE}}}_{{\rm{SP}}}\approx \frac{16.083-\,\max \,\mathrm{\{6.163},\mathrm{5.243\}}}{\max \,\mathrm{\{6.163},\mathrm{5.243\}}}\times 100=\mathrm{160.96[ \% ].}$$

and

$${{\rm{CRE}}}_{{\rm{SP}}}^{-}\approx \frac{16.083-7.484}{7.484}\times 100=\mathrm{114.90[ \% ].}$$

The results are quite clearcut. For this neuron, replacing CRE_SP_ by CRE^−^_SP_ corresponds to a drop from about 161% to about 115% with spontaneous activity removed, and from about 138% to about 171% when spontaneous activity was retained. Thus, applying the new index may well lead to dropping the “multisensory” label for this neuron depending, of course, on one’s criterion for attaching that label.

### Computing CRE^−^_SP_ and CRE_SP_ for data from 20 neurons

The total data set comprised 84 recording blocks from 20 SC cells (15 stimulus presentations in each block), where the number of spikes to visual-auditory stimulation was found significantly larger than the maximum of responses to unisensory stimulation, according to categorization from the Wallace lab. In 57 of these blocks, there was no response at all from one of the unisensory modalities (either visual or auditory) but a significant response increase for bimodal stimulation. For those cases, although considered as manifestation of multisensory integration, we have CRE^−^_SP_ = CRE_SP_ by definition, so the comparison is void. The data from the remaining 27 recording blocks were available for comparing both indexes.

**Figure 1:**
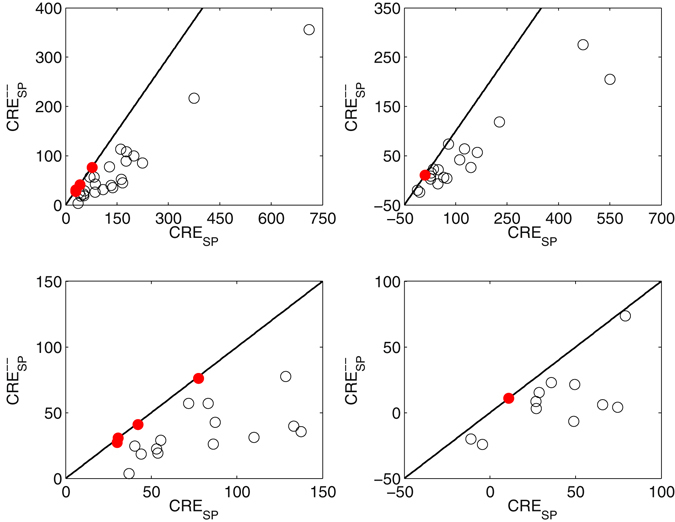
Pairs of sample estimates of (CRE_SP_, CRE^−^_SP_) based on 27 recording blocks (15 stimulus presentations in each block). In the left-hand panels spontaneous activity was included, in the right-hand panels it has been removed (see caption of Table 2). The lower panels display details of the upper ones, for better visibility. Filled circles (red) indicate no significant difference between CRE_SP_ and CRE^−^_SP_, based on bootstrap confidence intervals, too small to be shown (*N* = 10,000, 1−α = 0.95). Thus, each open circle refers to a recording where the label “multisensory” may be lost when applying the new measure. There were 4 out of 27 cases with no significant difference between both measures (left panels), after spontaneous activity was removed, only 1 out of 19 cases was not significant (right panels). In the latter, the number of possible comparisons decreased to 19 because in the other blocks there was no activity left for one of the unisensory conditions.

The points of Fig. 1 depict pairs of sample estimates of (CRE_SP_, CRE^−^_SP_), with spontaneous activity retained in the two left panels and removed in the two right panels (for details of the recording procedure see caption of Table 2). In order to obtain confidence interval estimates for the difference between CRE^−^_SP_ and CRE_SP_, each of the 27 blocks underwent a bootstrap procedure, i.e., 10,000 random samples of *N* = 15 were taken with replacement from the sets of spike frequencies for visual (**V**), auditory (**A**), and bimodal (**VA**) stimulation. For each sample, both CRE^−^_SP_ and CRE_SP_ were computed yielding a 95% confidence interval for their difference in each of the 27 recording blocks. There were 4 out of 27 cases with no significant difference between both measures (left panels, filled (red) circles), after spontaneous activity was removed, only 1 out of 19 cases was not significant (right panels). In the latter, the number of possible comparisons decreased to 19 because in the other blocks there was no activity left for one of the unisensory conditions.

In summary, there is a significant drop going from CRE_SP_ to CRE^−^_SP_ for most recording blocks. Whether or not the label “multisensory” is actually lost, however, will depend on the criteria of the statistical test comparing the sample means.

## Discussion

The issue of how to quantify crossmodal response enhancement due to the occurrence of multisensory integration has been under discussion in both behavioral and neurophysiological research. The most widely used index up to now expresses crossmodal enhancement as a proportion of the strongest unisensory response. It has descriptive value but lacks a theoretical basis. Such a foundation is essential because, as widely acknowledged in both reaction time and neural studies, being responsive to multiple sensory modalities does not guarantee that the response has been generated by actually integrating the multiple sensory inputs, rather than simply responding to the most salient stimulus modality. Here we suggest a new index that measures by how much the multisensory response surpasses the level obtainable by optimally combining the unisensory responses. Optimality is defined by referring to a probability summation mechanism that combines the unisensory responses with maximal negative dependence. Importantly, no claim is made that the system actually operates under this mechanism, it only serves as well-defined benchmark against which to gauge the crossmodal response.

In order to prevent misunderstandings, it may be useful to expand a bit on the statistical context of the new index. Operationally, classifying a neuron as “multisensory” refers to the outcome of a statistical test: the number of spikes of the neuron is significantly higher (or lower, in case of inhibition) under crossmodal stimulation than under unimodal stimulation (see, e.g., review paper bei Stein and colleagues^33^, p. 521 “Whether considering neural signals or behavioural performance, this [multisensory integration] is defined operationally as a statistically significant difference between the response evoked by a cross-modal combination of stimuli and that evoked by the most effective of its components individually”). This is routine procedure for the traditional index and applies to the new index as well. The null hypothesis is that a given neuron is not multisensory; the alternative being that the neuron is actually of the multisensory type. For both indexes, the criterion for classifying a neuron as “multisensory” is a statistically significant deviation of the observed number of spikes under crossmodal stimulation from that null hypothesis. What our measure–as well as the traditional one–yields is a (numerical) definition of the null hypothesis. The new measure is more conservative than the traditional one in the following sense: because the numerical value of the null hypothesis corresponding to the new measure is located above (to the right of) the one for the traditional measure, the *p*-value, obtained for a given sample and keeping everything else fixed, tends to be larger than the *p*-value for the traditional measure (note that this description is at the level of the expected number of spikes, not the CRE indexes).

It has been demonstrated here that the new index can be defined in a unified manner for studying both reaction times and responses by single neurons (spike frequencies). Whereas the index is closely related to the race model inequality, a widely used testing procedure for multisensory integration in reaction times, its application to neural responses has new and potentially important consequences: neurons previously labeled as “multisensory” may lose that property since the new index tends to yield smaller values for the amount of crossmodal enhancement. This was exemplified here with a data set collected from single SC neurons. The extent to which this holds more generally can only be determined by a large-scale investigation of a multitude of neurons from empirical studies. Obviously, at the level of a (sub-)population of neurons, such a relabeling may lead to a reassessment of the distribution of multisensory neurons and different types of unisensory neurons for that region.

In this context, despite the complete mathematical analogy between applying the race model inequality and using the new index, it is worth pointing out a subtle difference concerning possible consequences. In case the race model is not violated, the typical conclusion is that “true” multisensory integration (often termed “coactivation”) may still have occurred, but not a level that could not be explained just as well by a simple probability summation mechanism. In the neuron case, when the observed crossmodal spike number is not statistically higher than predicted by new index, the label “multisensory” will be removed if it was present under the traditional index. It would be good practice to point out that this relabeling is based on not being able to reject the null hypothesis based on probability summation with maximally negative dependency. Moreover, studies probing the entire scope of the behavior of multisensory neurons, e.g. by looking at intrinsic differences in the dynamic range of these neurons (see ref. 8), may come to different conclusion when using the new index.

We showed that the new index, CRE^−^_SP_, is easy to compute and does not require any specific assumption about the spike distribution. Given that “single neuron responses in both unisensory and multisensory brain regions are often perplexing, temporally complex and can be counterintuitive to expectations”^34^, probability summation cannot be ruled out as processing mode of a single neuron, and negative dependency would occur, e.g., under a neuron’s fixed limited capacity to process two modalities at the same time. The special case of Poisson-distributed spikes demonstrated that the new index is sensitive to variability in the data, in contrast to the traditional index which by definition only depends on the means of the uni- and multisensory response distributions. This is important because it shows that a probability mechanism can even mimic the “inverse effectiveness” rule often seen as a marker for multisensory integration.

Future research should address a number of issues. For example, it should be straightforward to generalize the new index to also capture crossmodal *inhibition*. Another issue is whether the logic of the new index can be extended to more than two modalities. Such a generalization is not obvious given that maximal negative dependence among three random variables is strongly limited. On a broader level, it would be interesting to explore whether the new index, or at least its logic, could be utilized beyond the level of single neuron responses, possibly including data from functional magnetic resonance studies^35^. As the authors of a recent review^36^ put it, “…, an enhanced BOLD response for multisensory relative to unisensory stimulation can be due to “true” multisensory neurons integrating stimulation from two or more sensory modalities, but it can just as well be explained by driving two unisensory sub-populations instead of one. If the latter scenario would be true, one might wrongly infer multisensory integration at the neuronal level.” Given the recent results by Miller *et al*.^11^, showing “… that the integration of temporally displaced sensory responses is also highly dependent on the relative efficacies with which they drive their common target neuron”, one may more generally question the usefulness of any static measure of crossmodal enhancement, and this may lead to add a temporal dimension to any quantitative index of crossmodal enhancement.

Last but not least, the methodological approach suggested here may also enable one to derive more specific information about the integration mechanism of multisensory neurons. A common categorization of such mechanisms is into “super-additive”, “additive”, and “sub-additive”, depending on how the unisensory activations combine to produce the multisensory response^5, 6^. The new index, CRE^−^_SP_, measures how far the observed multisensory response is above a particular sub-additive combination rule, i.e., the maximum rule under negative dependency. Interestingly, from^37^ (and more recent papers in actuarial statistics), it is possible to compute the expected value of the maximally achievable sum of two random variables under negative dependency. The resulting value of a (modified) CRE^−^_SP_ for the sum can then be used to gauge, e.g., how far an observed super-additive response is away from a simple additive (linear) combination of the unisensory responses.

## References

[1] Stein, B. (ed.) The New Handbook of multisensory processing (MIT Press, 2012).

[2] Stein, B. & Meredith, M. *The merging of the senses* (MIT Press, 1993).

[3] Stein B (2010). Semantic confusion regarding the development of multisensory integration: a practical solution. European Journal of Neuroscience.

[4] Stein B, Stanford T, Ramachandran R, Perrault T, Rowland B (2009). Challenges in quantifying multisensory integration: alternative criteria, models, and inverse effectiveness. Experimental Brain Research.

[5] Stevenson R (2014). Identifying and quantifying multisensory integration: a tutorial review. Brain Topography.

[6] Stanford T, Quessy S, Stein B (2005). Evaluating the operations underlying multisensory integration in the cat superior colliculus. Journal of Neuroscience.

[7] Meredith M, Stein B (1983). Interactions among converging sensory inputs in the superior colliculus. Science.

[8] Perrault T, Vaughan J, Stein B, Wallace M (2005). Superior colliculus neurons use distinct operational modes in the integration of multisensory stimuli. Journal of Neurophysiology.

[9] Populin, L. & Yin, T. Bimodal interactions in the superior colliculus of the behaving cat. *Journal of Neuroscience* **22**, 2826–2834, 20026231 (2002). 10.1523/JNEUROSCI.22-07-02826.2002 PMC6758283 11923447

[10] Beauchamp MS (2005). Statistical criteria in fMRI studies of multisensory integration. Neuroinformatics.

[11] Miller R, Pluta S, Stein B, Rowland B (2015). Relative unisensory strength and timing predict their multisensory product. Journal of Neuroscience.

[12] Wallace, M. Personal communication. July 18, 2015 (2015).

[13] Cappe, C., Murray, M. M., Barone, P. & Rouiller, E. Multisensory facilitation of behavior in monkeys: effects of stimulus intensity. *Journal of Cognitive Neuroscience* (2009). 10.1162/jocn.2010.21423 20044892

[14] van der Stoep, N., van der Stigchel, S., Nijboer, T. & van der Smagt, M. Audiovisual integration in near and far space: effects of changes in distance and stimulus effectiveness. *Experimental Brain Research* (2015). 10.1007/s00221-015-4248-2 PMC4828496 25788009

[15] Diederich A, Colonius H (2004). Bimodal and trimodal multisensory enhancement: effects of stimulus onset and intensity on reaction time. Perception and Psychophysics.

[16] Buchholz V, Goonetilleke SC, Medendorp W, Corneil B (2012). Greater benefits of multisensory integration during complex sensorimotor transformations. Journal of Neurophysiology.

[17] Raab D (1962). Statistical facilitation of simple reaction time. Transactions of the New York Academy of Sciences.

[18] Miller JO (1982). Divided attention: Evidence for coactivation with redundant signals. Cognitive Psychology.

[19] Miller JO (2016). Statistical facilitation and the redundant signals effect: What are race and coactivation models?. Attention, Perception, and Psychophysics.

[20] Luce, R. Response times: Their role in inferring elementary mental organization (Oxford University Press, New York, NY, 1986).

[21] Colonius H (1990). Possibly dependent probability summation of reaction time. Journal of Mathematical Psychology.

[22] Gondan M, Minakata K (2016). A tutorial on testing the race model inequality. Attention, Perception, and Psychophysics.

[23] Colonius H, Diederich A (2006). Race model inequality: Interpreting a geometric measure of the amount of violation. Psychological Review.

[24] Thorisson, H. *Coupling, stationarity, and regeneration* (Springer Verlag, New York, NY, 2000).

[25] Ross, S. *Stochastic processes* (John Wiley & Sons, New York, NY, Second edn, 1996).

[26] Miller J (1986). Timecourse of coactivation in bimodal divided attention tasks. Perception & Psychophysics.

[27] Colonius, H. An invitation to coupling and copulas, with applications to multisensory modeling. *Journal of Mathematical Psychology*, doi:10.1016/j.jmp.2016.02.004 (2016).

[28] Fréchet M (1951). Sur les tableaux de corrélation dont les marges sont donnés. Annales de l’Université de Lyon Section A, Séries.

[29] Joe, H. *Multivariate models and dependence concepts*. No. 73 in Monographs on Statistics and Applied Probability (Chapman & Hall, London, UK, 1997).

[30] Johnson, N., Kotz, S. & Kemp, A. *Univariate discrete distributions*. Wiley Series in Probability and Mathematical Statistics (John Wiley & Sons, New York, NY, second edn, 1992).

[31] Griffiths R, Milne R, Wood R (1979). Aspects of correlation in bivariate Poisson distributions and processes. Australian Journal of Statistics.

[32] Ghose D, Barnett Z, Wallace M (2012). Impact of response duration on multisensory integration. Journal of Neurophysiology.

[33] Stein B, Stanford T, Rowland B (2014). Development of multisensory integration from the perspective of the individual neuron. Nature Reviews Neuroscience.

[34] Chandrasekaran C (2017). Computational principles and models of multisensory integration. Current Opinion in Neurobiology.

[35] Klemen J, Chambers CD (2012). Current perspectives and methods in studying neural mechanisms of multisensory interactions. Neuroscience and Biobehavioral Reviews.

[36] Goebel R, van Atteveldt N (2009). Multisensory functional magnetic resonance imaging: a future perspective. Experimental Brain Research.

[37] Rüschendorf L (1982). Random variables with maximum sums. Advances in Applied Probability.

